# Neuromuscular blocking agents in acute respiratory distress syndrome: updated systematic review and meta-analysis of randomized trials

**DOI:** 10.1186/s40635-020-00348-6

**Published:** 2020-10-23

**Authors:** Nehal Tarazan, Moayad Alshehri, Sameer Sharif, Zainab Al Duhailib, Morten Hylander Møller, Emilie Belley-Cote, Mohammed Alshahrani, John Centofanti, Lauralyn McIntyre, Bandar Baw, Maureen Meade, Waleed Alhazzani, for the GUIDE Group

**Affiliations:** 1 Department of Medicine, McMaster University, Hamilton, Canada; 2 Department of Internal Medicine, King Saud University, Riyadh, Saudi Arabia; 3 Department of Medicine, Division of Emergency Medicine, McMaster University, Hamilton, Canada; 4 Department of Critical Care Medicine, King Faisal Specialist Hospital and Research Centre, Riyadh, Saudi Arabia; 5 Department of Intensive Care 4131, Copenhagen University Hospital Rigshospitalet, Copenhagen, Denmark; 6 Department of Emergency and Critical Care, Imam Abdulrahman Ben Faisal University, Dammam, Saudi Arabia; 7 Department of Medicine, University of Ottawa, Ottawa, ON Canada; 8 Clinical Epidemiology Program, Ottawa Hospital Research Institute, Ottawa, ON Canada; 9 Department of Health Research Methods, Evidence and Impact, McMaster University, Hamilton, ON L8S 4K1 Canada

**Keywords:** ARDS, Neuromuscular blockade, Systematic review

## Abstract

**Purpose:**

Existing clinical practice guidelines support the use of neuromuscular blocking agents (NMBA) in acute respiratory distress syndrome (ARDS); however, a recent large randomized clinical trial (RCT) has questioned this practice. Therefore, we updated a previous systematic review to determine the efficacy and safety of NMBAs in ARDS.

**Methods:**

We searched MEDLINE, EMBASE (October 2012 to July 2019), the Cochrane (Central) database, and clinical trial registries (ClinicalTrials.gov, ISRCTN Register, and WHO ICTRP) for RCTs comparing the effects of NMBA as a continuous infusion versus placebo or no NMBA infusion (but allowing intermittent NMBA boluses) on patient-important outcomes for adults with ARDS. Two independent reviewers assessed the methodologic quality of the primary studies and abstracted data.

**Results:**

Seven RCTs, including four new RCTs, met eligibility criteria for this review. These trials enrolled 1598 patients with moderate to severe ARDS at centers in the USA, France, and China. All trials assessed short-term continuous infusions of cisatracurium or vecuronium. The pooled estimate for mortality outcomes showed significant statistical heterogeneity, which was only explained by a subgroup analysis by depth of sedation in the control arm. A continuous NMBA infusion did not improve mortality when compared to a light sedation strategy with no NMBA infusion (relative risk [RR] 0.99; 95% CI 0.86–1.15; moderate certainty; *P =* 0.93). On the other hand, continuous NMBA infusion reduced mortality when compared to deep sedation with as needed NMBA boluses (RR 0.71; 95% CI 0.57–0.89; low certainty; *P =* 0.003). Continuous NMBA infusion reduced the rate of barotrauma (RR 0.55; 95% CI 0.35–0.85, moderate certainty; *P* = 0.008) across eligible trials, but the effect on ventilator-free days, duration of mechanical ventilation, and ICU-acquired weakness was uncertain.

**Conclusions:**

Inconsistency in study methods and findings precluded the pooling of all trials for mortality. In a pre-planned sensitivity analysis, the impact of NMBA infusion on mortality depends on the strategy used in the control arm, showing reduced mortality when compared to deep sedation, but no effect on mortality when compared to lighter sedation. In both situations, a continuous NMBA infusion may reduce the risk of barotrauma, but the effects on other patient-important outcomes remain unclear. Future research, including an individual patient data meta-analysis, could help clarify some of the observed findings in this updated systematic review.

## Take home message

The current body of evidence does not support the routine and early use of a continuous NMBA infusion in all patients with ARDS, intermittent NMBA boluses is probably adequate for most patients. However, in patients with severe ARDS who are deeply sedated, the use of a continuous NMBA is a reasonable option.

## Introduction

Acute respiratory distress syndrome (ARDS) is a life-threatening condition that complicates a variety of critical illnesses, including sepsis, pneumonia, and trauma [1]. In a recent international observational study involving 29,144 patients, 10% of all patients admitted to the intensive care unit (ICU) and 23% of mechanically ventilated patients had ARDS [2]. Importantly, the mortality among patients with severe ARDS was 46.1% [2]. Patients who survive ARDS are at high risk for cognitive decline, depression, post-traumatic stress disorder, and persistent muscular weakness [3, 4].

The priorities in the care of patients with ARDS are identifying and treating the underlying cause, along with supportive therapies to prevent further lung injury. Most recent advances in the treatment of ARDS focus on the latter, i.e., minimizing ventilator-associated lung injury through the application of low tidal volumes, high levels of positive end-expiratory pressure (PEEP), prone ventilation, and neuromuscular blockade [5–7].

Of the pharmacologic treatment options for adults with ARDS, only neuromuscular blocking agents (NMBAs) have demonstrated a mortality benefit in patients with severe ARDS [8]. Previous trials showed that a continuous NMBA infusion for 48 h improves survival in patients with moderate to severe ARDS [8]. The mechanism of this benefit may be multifaceted, but likely involves a reduction in patient–ventilator desynchrony and, in turn, ventilator-associated lung injury [9]. NMBAs also reduce the work of breathing, and may reduce the accumulation of alveolar fluid [10], decrease pulmonary and systemic inflammation, and decrease oxygen consumption [10]. A multicenter observational study from centers in Canada and Saudi Arabia suggested that 42% of critically ill adults with moderate to severe ARDS receive NMBA therapy [11].

Results of a recent multicenter clinical trial have challenged current practice recommendations. The Reevaluation of Systemic Early Neuromuscular Blockade (ROSE) trial randomized 1006 patients with moderate or severe ARDS to receive either deep sedation with a continuous cisatracurium infusion (an NMBA), or lighter sedation with short intermittent NMBA infusions only when deemed necessary by the attending physicians [12]. The trial stopped early for perceived futility [12]. Given these results and the possible publication of other RCTs, we undertook an updated systematic review and meta-analysis to clarify the effects of NMBAs on patient-important outcomes for adults with ARDS. The results of this systematic review will help inform practice guidelines [13].

## Methods

This review followed an internal protocol and adhered to the *Preferred Reporting Items for Systematic Reviews and Meta-Analyses* (PRISMA) reporting guidelines [14].

### Eligibility criteria

Eligible studies met all of the following criteria: (1) the design was a parallel-group RCT; (2) the population was adults with ARDS of any severity; (3) the intervention included any continuous NMBA infusion, at any dose or duration, compared to placebo or no continuous NMBA infusion but allowing the use of as needed NMBA boluses; (4) outcomes included any of: mortality at 28 days, ICU discharge, or hospital discharge (truncated at 90 days); long-term outcomes (physical function at 3 months; quality of life at 3 months; cognitive function at 3 months); ICU-acquired weakness; duration of mechanical ventilation; ventilator-free days (VFDs); ICU or hospital length of stay; barotrauma (including pneumothorax, pneumomediastinum, pneumatocele, or subcutaneous emphysema); or changes in oxygenation measured by using the ratio of arterial oxygen partial pressure to fractional inspired oxygen (PO_2_/FiO_2_ ratio).

### Search strategy

We updated our prior search strategy and electronically searched MEDLINE, EMBASE (October 2012 to July 2019), the Cochrane (Central) database, and clinical trial registries (ClinicalTrials.gov, ISRCTN Register, and WHO ICTRP). The search strategy can be found in the electronic supplementary material (ESM).

### Study selection and data extraction

Two reviewers independently screened titles and abstracts in duplicate. The same reviewers assessed the full-text of potentially eligible articles and abstracted relevant data from eligible studies. A third reviewer resolved disagreements between reviewers.

### Assessment of the risk of bias of included studies

For each study, reviewers used the Cochrane Handbook Risk of Bias tool to judge the adequacy of randomization, concealment, blinding, and outcome-data completeness, and to check for selective outcome assessment and other possible sources of bias [15]. They judged the risk of bias in each of these domains as high, low, or unclear. The overall risk of bias for an individual study was categorized as low when the risk of bias was low in all domains; unclear when the risk of bias was unclear in at least one domain, with no high-risk domains; or high when the risk of bias was high in at least one domain.

### Statistical analysis

We used RevMan 5.3 software to perform the analyses. If appropriate, we pooled the effect estimates across studies using a random-effects model with Mantel-Haenszel weighting and the methods of DerSimonian and Laird [16]. If less than 3 RCTs contributed to the analysis, we used a fixed effect model instead. We generated summary estimates of relative risk (RR) for dichotomous outcomes and mean differences (MDs) for continuous outcomes, each with associated 95% confidence intervals (CIs). We assessed for heterogeneity between studies using the Chi^2^ statistic (*P* < 0.01 indicating substantial heterogeneity) and the *I*^2^ statistic (> 50% indicating substantial heterogeneity), and by inspecting forest plots. We identified less than 10 studies; therefore, we did not use funnel plots or conventional statistical methods to assess for publication bias [17].

We stipulated a number of pre-planned, a priori exploratory analyses to assess potential reasons for differing results (if any) across studies. We hypothesized that the following factors might generate estimates of greater benefit: high or unclear risk of bias (versus low), more severe hypoxemia at baseline (PO_2_/FiO_2_ < 100, versus 100 to 200), targeting deep sedation in the control group (versus light sedation) as defined by individual studies, high PEEP strategy as defined by individual studies (versus low PEEP), early (within 48 h of intubation) initiation of NMBA infusion (versus late), the use of prone ventilation (versus not), duration of NMBA infusion (< 48 h versus > 48 h), and the cause of ARDS (sepsis versus non-sepsis related ARDS).

When significant statistical heterogeneity was present in the pooled analysis and was explained by a subgroup analysis, we reported the subgroup estimates separately as the primary results.

### Certainty of the evidence

We used the *Grading of Recommendations Assessment, Development and Evaluation* (GRADE) approach to assess the certainty of evidence for each outcome [18]. Reviewers assessed the impact of risk of bias, inconsistency, indirectness, imprecision, and publication bias on the certainty of the evidence. The certainty of evidence can be classified as very low, low, moderate, or high.

## Results

**Fig. 1:**
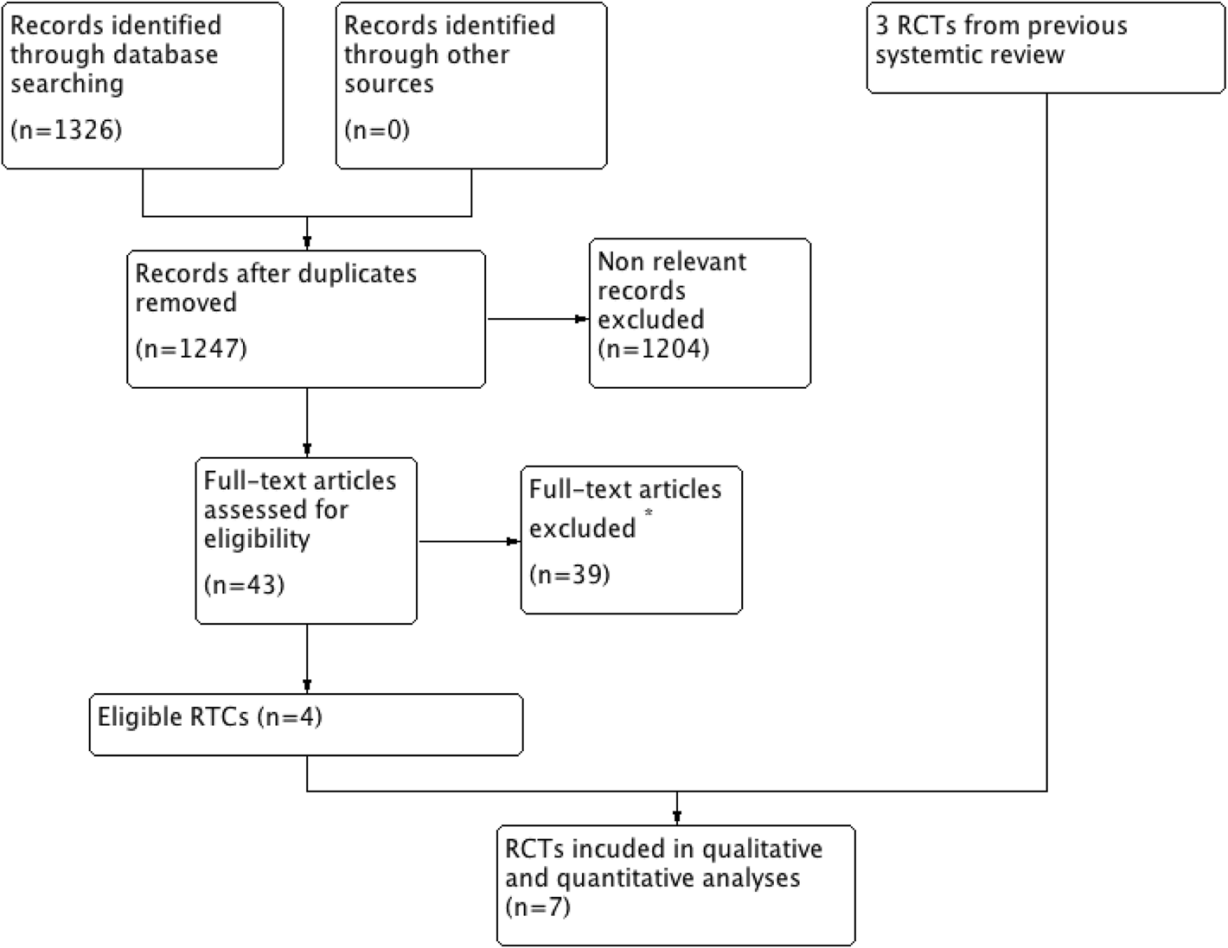
Summary of evidence search and selection. Flow diagram showing steps of study selection

After screening 1247 titles and abstracts, reviewers assessed 43 full-texts for eligibility. Four new trials were eligible for this review (Fig. 1), resulting in a total of 7 trials (1598 patients) [9, 12, 19–23]. Of the new trials, the largest was conducted in the USA (*N* = 1006), with one in France (*N* = 24), and the other two in China (*N* = 41 and *N* = 96). The former two trials studied cisatracurium, and the latter two studied vecuronium.

All seven studies were specifically designed to investigate the effects of a continuous NMBA infusion on gas exchange, inflammatory markers, and/or clinical outcomes in patients with ARDS. Four studies used a 48-h infusion of cisatracurium [9, 12, 19, 20], whereas the other three studies did not pre-specify a duration for NMBA infusions. Weight-based dosing of cisatracurium was used in two of the studies [19, 20], and a fixed high dose was used in another three studies (15 mg bolus, followed by a continuous infusion of 37.5 mg per hour) [9, 12, 22]. The two studies of vecuronium used the following maintenance doses, without boluses being reported: 0.05 mg/kg/h and 1 μg/kg/min [21, 23]. All studies reported 28-day mortality; other reported outcomes varied by study.

The interventions used in the control arm varied between studies; three studies used a 48-h infusion of placebo (normal saline) with deep sedation, an additional three studies did describe the control they used, and one study used light sedation in the control group. Five studies allowed the use of NMBA boluses as needed in the control group [9, 12, 19, 20, 22]. The proportion of patients receiving NMBA boluses in the control group ranged between 14.3% and 29% across studies.

Deep sedation was defined as a Ramsay score of 6, and light sedation was defined as a Ramsay score of 2–3 [12], Richmond Agitation-Sedation Scale (RASS) score of 0 to − 1, and/or a Riker score of 3 to 4. In total, 257 patients included in this review received concomitant corticosteroid therapy during the study period of NMBA infusion. The characteristics of the included studies are presented in Table 1.

**Table 1:** Characteristics of included studies

Study	Population	Agent/Dose	NMBA PRN	PEEP	Prone ventilation	Sedation target	Sedative	Ventilation Protocol	Steroids
**Gainnier 2004** **[**18**]**	ARDS PaO_2_/FiO_2_ < 150 PEEP ≥ 5 Eligible < 36 h Exclude prior NMBA	**cisatracurium** bolus of 50mg then infusion at 5 mcg/kg/min	NR	11.1±2.8	14.3%	Deep Ramsay score 6	Midazolam Sufentanil	Yes	7.1%
		Placebo (0.9% NS)	48 h: 7.1%	10.9±2.4	14.3%	Deep Ramsay score 6	Midazolam Sufentanil	Yes	14.3%
**Forel 2006** **[**19**]**	ARDS Intubated < 48 h PaO_2_/FiO_2_ < 200 Exclude recent steroids or NMBA	**cisatracurium** bolus of 0.2 mg/kg then infusion at 5 mcg/kg/min Titrated (increase of 20% if TOF >1) to obtain no response on TOF	48 h: NR ICU stay: 5.6%	9±2.3	-	Deep Ramsay score 6	Midazolam Sufentanil	Yes Same protocol for the first 5 days	5.5%
		Placebo (0.9%NS)	48 h: 0% ICU stay: 5.6%	9.9±2.9	-	Deep Ramsay score 6	Midazolam Sufentanil	Yes	0%
**Papazian 2010** **[**8**]**	ARDS PaO_2_/FiO_2_ < 150 Eligible < 48 h Exclude prior NMBA	**cisatracurium** 15 mg bolus then infusion of 37.5 mg/h for 48h	48 h: 10% ICU stay: 50%	9.2±3.2	28%	Deep Ramsay score 6	Sufentanil Midazolam Ketamine Propofol	Yes	39.5%
		Placebo	48 h: 22% ICU stay: 56%	9.2±3.5	29%	Deep Ramsay score 6	Sufentanil Midazolam Ketamine Propofol	Yes	45.1%
**Lyu 2014** **[**20**]**	Severe sepsis and moderate to severe ARDS by Berlin criteria	Vecuronium	-	-	-	Deep	-	-	-
		No placebo	-	-	-	Deep	-	-	-
**Rao 2016** **[**22**]**	ARDS by Berlin criteria	Vecuronium	-	-	-	-	-	-	-
		No placebo	-	-	-	-	-	-	-
**Guervilly 2016** **[**21**]**	ARDS; PaO_2_/FiO_2_ < 150 with PEEP at least 5 within 48 hours of ARDS onset	**cisatracurium** 15 mg bolus then infusion of 37.5 mg/h for 48h	-	10 (9; 12)	-	Deep Ramsay score 6	Sufentanil Midazolam Ketamine	ARDSnet protocol	-
		No infusion, but severe ARDS received PRN NMBA	-	11(10;11.5)	-	Deep Ramsay score 6	Sufentanil Midazolam Ketamine	ARDSnet protocol	-
**ROSE 2019** **[**11**]**	ARDS with PaO_2_/FiO_2_ <150 + PEEP>8cmH2O	**cisatracurium** 15 mg bolus then infusion of 37.5 mg/h for 48h	48 h: 0.9% After 48h: 16.0%	12.6±3.6	16.8%	Deep RASS -5 or Ramsay 5-6	-	ARDSNet	Day 7: 17.0%
		Usual care	48h:17.0% After 48h: 7.9%	12.5±3.6	14.9%	Light RASS: 0 to -1, Riker: 3 to 4, or Ramsay: 2-3	-	ARDSNet	Day 7: 16.4%

*ARDS* acute respiratory distress syndrome, *ARMA* the Acute Respiratory Distress Syndrome Network, *ICU* intensive care unit, *NMBA* neuromuscular blocking agents, *NS* normal saline, *PaO*_*2*_*/FiO*_*2*_ ratio of partial arterial pressure of oxygen to fraction of inspired oxygen, *PEEP* positive end expiratory pressure, *RASS* Richmond Agitation-Sedation Scale, *TOF* train of four

All studies were judged to be at low risk of bias except 2 that were at high risk of attrition and reporting bias [21, 23].

**Fig. 2:**
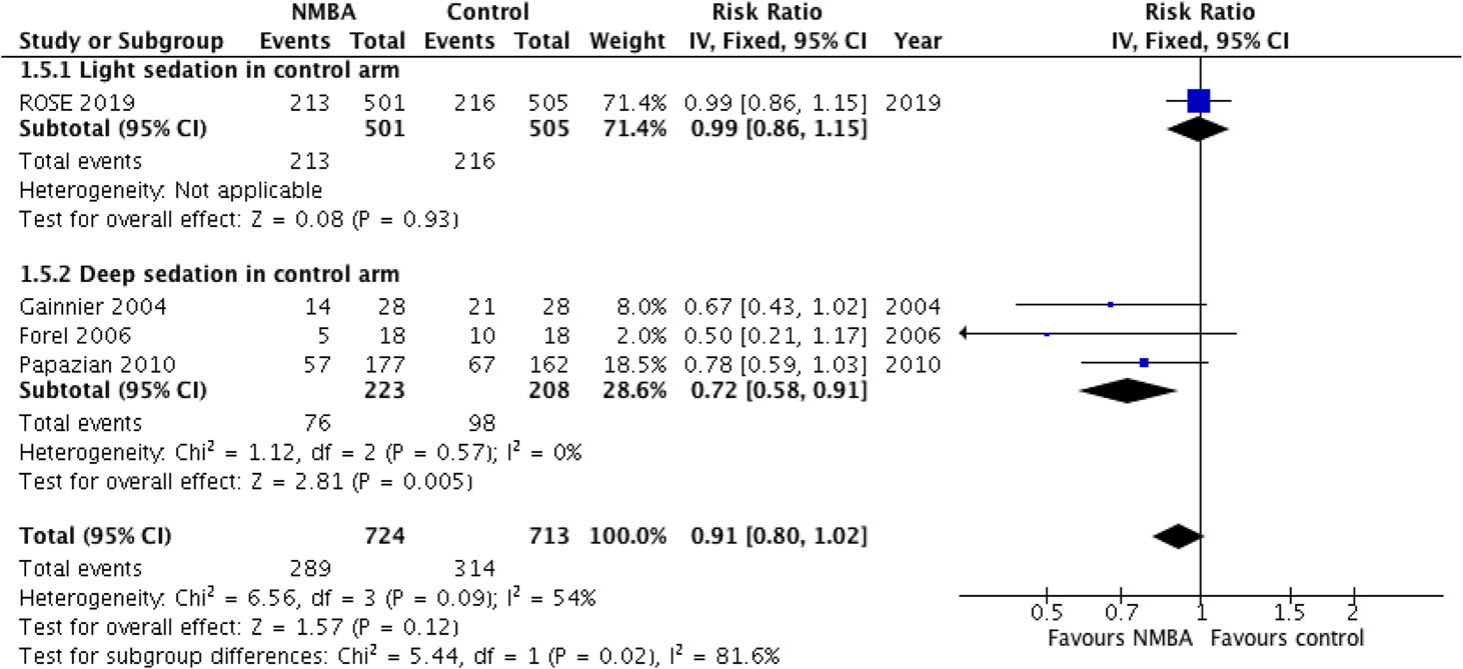
Forest plot for mortality outcome by subgroups of sedation depth in the control arm;results are shown by using random-effects model with relative risk and 95% confidence interval. NMBA, neuromuscular blocking agents; CI, confidence interval

Seven studies (1598 patients) reported on mortality and five reported the depth of sedation. Of note, one trial reported 21-day mortality; we included this trial in the analysis of 28-day mortality as both time-points reflect a short-term mortality outcome that is fairly similar [21]. The use of NMBA infusion was associated with lower 28-day mortality (RR 0.74; 95% CI 0.56–0.98; *I*^*2*^ = 45%; low certainty; *P =* 0.03). However, the effect on 90-day mortality was not statistically significant (RR 0.78, 95% CI 0.60–1.01, *I*^*2*^ = 55%; low certainty; ESM; *P =* 0.06). Due to significant clinical and statistical heterogeneity, we have more certainty in the effect estimates from the subgroup analysis of light versus deep sedation; therefore, the analysis separating studies by depth of sedation are the more trustworthy estimates for mortality outcomes (Fig. 2).

**Fig. 3:**
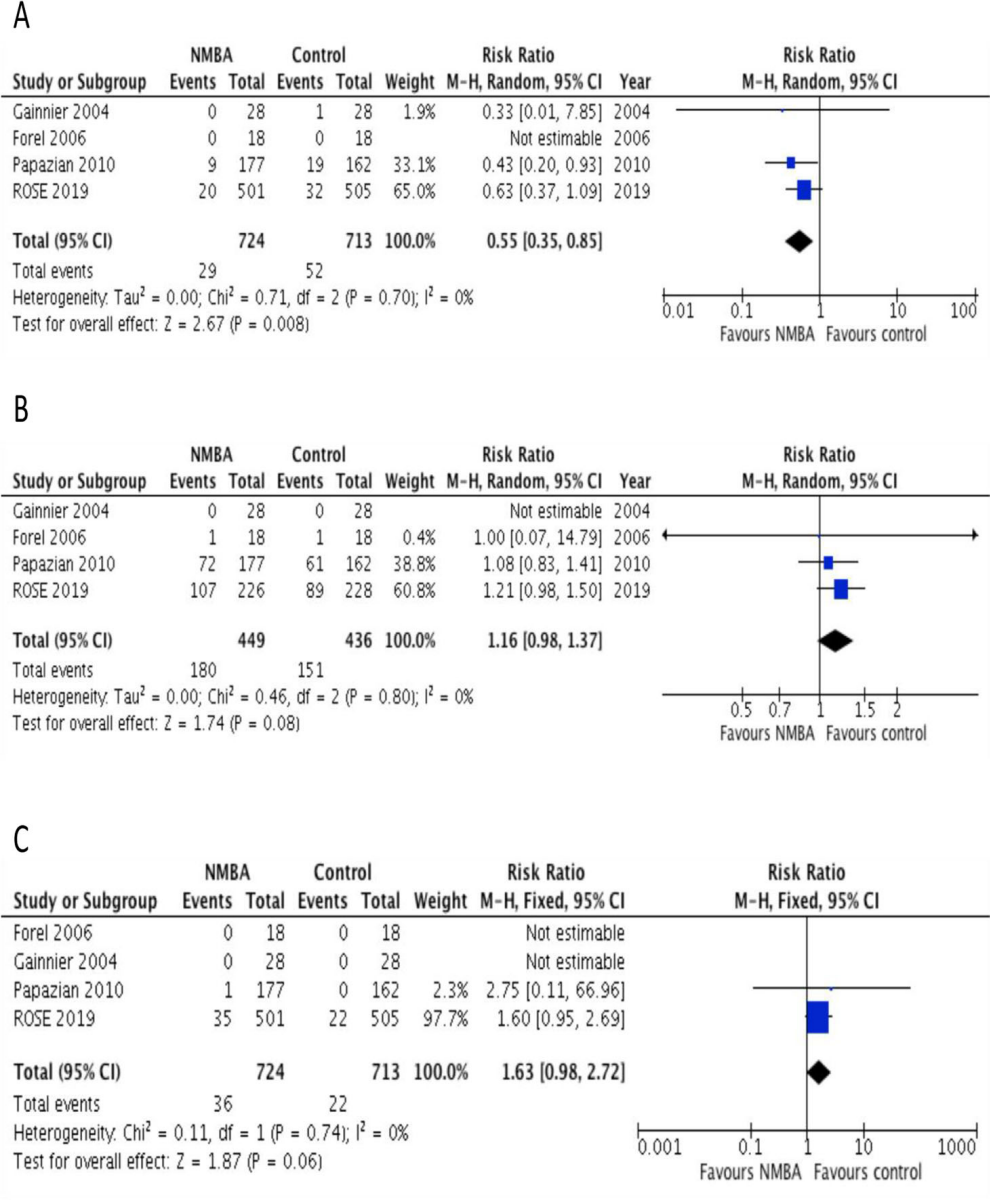
Forest plot A comparing NMBA infusion to placebo or usual care for barotrauma; Forest plot B comparing NMBA infusion to placebo or usual care for ICU-acquired weakness; Forest plot C comparing NMBA infusion to placebo or usual care for adverse events. NMBA, neuromuscular blocking agents; CI, confidence interval; ICU, intensive care unit

NMBA infusion reduced the risk of barotrauma (RR 0.55; 95% CI 0.35–0.85, *I*^*2*^ = 0%; moderate certainty; *P =* 0.008; Fig. 3), but did not affect VFD at 28 days (MD 0.57; 95% CI–0.43, 1.57, *I*^*2*^ = 0%, *P* = 0.46; low certainty) or the duration of mechanical ventilation (MD − 1.21; 95% CI − 4.23, 1.81; *I*^*2*^ = 0%; low certainty; *P =* 0.43; ESM). Only one RCT reported ICU length of stay, and this did not differ between the two groups (MD − 1.80 days; 95% CI − 5.93–2.33; *P =* 0.39; low certainty).

The use of NMBA infusion may increase the risk of ICU-acquired weakness; however, the 95% CI included no difference (RR 1.16; 95% CI 0.98, 1.37, *I*^*2*^ = 0%; moderate certainty; *P =* 0.08; Fig. 3). The pooled analysis suggested better PaO_2_/FiO_2_ in the NMBA group at 24, 48, and 72 h, but only the result at 72 h was statistically significant (ESM). The ROSE trial reported on long-term outcomes; the use of NMBA did not improve long-term outcomes (Table 2).

**Table 2:** Summary of findings

Outcomes	№ of participants (studies) Follow-up	Certainty of the evidence (GRADE)	Relative effect (95% CI)	Anticipated absolute effects	
				Risk with no infusion (but intermittent as needed NMBA)*	Risk difference with an infusion of neuromuscular blockade
Hospital mortality subgroup (compared to light sedation)	1006 (1 RCT)	⨁⨁⨁◯ Moderate^a,b^	**RR 0.99** (0.86 to 1.15)	428 per 1000	**4 fewer per 1000** (60 fewer to 64 more)
Hospital mortality subgroup (compared to deep sedation)	431 (3 RCTs)	⨁⨁◯◯ Low^b,c^	**RR 0.72** (0.58 to 0.91)	471 per 1000	**132 fewer per 1000** (198 fewer to 42 fewer)
Mortality–28-day mortality (pooled for all trials)	1598 (7 RCTs)^d^	⨁⨁◯◯ Low^b,e,f^	**RR 0.74** (0.56 to 0.98)	369 per 1000	**96 fewer per 1000** (162 fewer to 7 fewer)
Mortality–hospital/90-day mortality (pooled for all trials)	1478 (5 RCTs)	⨁⨁◯◯ Low^b,g^	**RR 0.78** (0.60 to 1.01)	437 per 1000	**96 fewer per 1000** (175 fewer to 4 more)
Mental health at 6 months	267 (1 RCT)	⨁⨁◯◯ Low^b,h^	**RR 1.03** (0.69 to 1.55)	254 per 1000	**8 more per 1000** (79 fewer to 140 more)
Cognitive function (MOCA scores)	287 (1 RCT)	⨁⨁◯◯ Low^i,j^	–	The mean cognitive Function (MOCA Scores) was **22.8** points	MD **0.6 points lower** (1.71 lower to 0.51 higher)
Quality of life	401 (1 RCT)^k^	⨁⨁◯◯ Low^b,l,m^	–	The mean quality of life was **0.73** units	MD **0.07 units lower** (0.15 lower to 0.01 higher)
Adverse events	1437 (4 RCTs)	⨁⨁◯◯ Low^b,n^	**RR 1.63** (0.98 to 2.72)	31 per 1000	**19 more per 1000** (1 fewer to 53 more)
ICU-acquired weakness	885 (4 RCTs)	⨁⨁⨁◯ Moderate^b,o^	**RR 1.16** (0.98 to 1.37)	346 per 1000	**55 more per 1000** (7 fewer to 128 more)
Hospital/90-day mortality (subgroup of patients with ARDS and P/F > 100)	542 (4 RCTs)	⨁⨁◯◯ Low^b,p,q^	**RR 0.87** (0.71 to 1.06)	444 per 1000	**58 fewer per 1000** (129 fewer to 27 more)
Hospital/90-day mortality (subgroup of patients with ARDS and P/F≤100)	895 (4 RCTs)	⨁⨁◯◯ Low^b,r,s^	**RR 0.95** (0.82 to 1.11)	427 per 1000	**21 fewer per 1000** (77 fewer to 47 more)
Hospital/90-day mortality (sensitivity analysis using ROSE late use of NMBA)	975 (5 RCTs)	⨁◯◯◯ Very low^b,h,t^	**RR 0.78** (0.57 to 1.06)	431 per 1000	**95 fewer per 1000** (185 fewer to 26 more)
Barotrauma	1437 (4 RCTs)	⨁⨁⨁◯ Moderate^b,u^	**RR 0.55** (0.35 to 0.85)	73 per 1000	**33 fewer per 1000** (47 fewer to 11 fewer)
Ventilator-free days at 28 days	1487 (5 RCTs)	⨁⨁◯◯ Low^b,v,w^	–	The mean ventilator-free days at 28 days was **14** days	MD **0.72 days more** (0.44 fewer to 1.88 more)
PO_2_/FiO_2_ post-randomization–PO_2_/FiO_2_ at 24 h post-randomization	1267 (4 RCTs)	⨁⨁◯◯ Low^b,x,y^	–	The median PO2/FiO2 post-randomization -PO2/FiO2 at 24 h post-randomization was **120**	MD **7.76 higher** (3.74 lower to 19.27 higher)
PO_2_/FiO_2_ post-randomization–PO_2_/FiO_2_ at 72 h post-randomization	1011 (4 RCTs)	⨁⨁◯◯ Low^b,x,z^	–	The mean pO2/FiO2 post-randomization -PO2/FiO2 at 72 h post-randomization was **100**	MD **15.21 higher** (1.9 higher to 28.52 higher)

**GRADE Working Group grades of evidence**

**High certainty:** We are very confident that the true effect lies close to that of the estimate of the effect

**Moderate certainty:** We are moderately confident in the effect estimate: The true effect is likely to be close to the estimate of the effect, but there is a possibility that it is substantially different

**Low certainty:** Our confidence in the effect estimate is limited: The true effect may be substantially different from the estimate of the effect

**Very low certainty:** We have very little confidence in the effect estimate: The true effect is likely to be substantially different from the estimate of effect

*CI* confidence interval, *RR*, risk ratio, *MD* mean difference

*The risk in the intervention group (and its 95% confidence interval) is based on the assumed risk in the comparison group and the relative effect of the intervention (and its 95% CI)

**Explanations**

^a^We downgraded the certainty in the evidence by one level for serious imprecision; the CI included both small benefit and harm

^b^We were not able to assess for publication bias using traditional methods because we identified less than 10 studies

^c^We downgraded the certainty of evidence by two levels for very serious imprecision, the total number of events was small (174 events)

^d^7 RCTs reported this outcome, including Gainnier M, et al. Crit Care Med. 2004;32(1):113-9.; Forel JM, et al. Crit Care Med. 2006;34(11):2749-57.; Papazian L, et al. N Engl J Med. 2010;363(12):1107-16.; Lyu G, et al. Zhonghua Wei Zhong Bing Ji Jiu Yi Xue. 2014;26(5):325-9.; Guervilly C, et al. Intensive Care Med. 2017;43(3):408-18.; N Engl J Med. 2019;380(21):1997–2008

^e^We downgraded the certainty of evidence by two levels for very serious inconsistency, although the I2 was 45%, there is inconsistency between the results of the most recent and large RCT (ROSE Trial) and the rest of the studies, which was not explained by any of the subgroup analyses, difficulty in reconciling and explaining the differences in results have lead us to lower our certainty in the estimates by 2 levels

^f^The 7 RCTs reported 547 deaths which is enough for us to consider the pooled estimates precise

^g^We downgraded the certainty of evidence by two levels for very serious inconsistency, although the *I*^2^ was 55%, there is inconsistency between the results of the most recent and large RCT (ROSE Trial) and the rest of the studies, which was not explained by any of the subgroup analyses, difficulty in reconciling and explaining the differences in results have lead us to lower our certainty in the estimates by 2 levels

^h^We downgraded the certainty of evidence by two levels for very serious imprecision; the CI was very wide including both substantial benefit and harm

^i^We downgraded the certainty of evidence by one level for serious risk of bias; many patients who were randomized did not complete the assessment

^j^We downgraded the certainty of evidence by one level for serious imprecision, the sample size was small

^k^N Engl J Med. 2019;380(21):1997–2008

^l^We downgraded the certainty of evidence by one level for risk of bias; the outcome is subjective and the trial was unblinded

^m^We downgraded the certainty of evidence by one level for serious imprecision; the CI included both harm and benefit, and the number of patients who were included the analysis at 3 months is small (< 50% of the original sample size)

^n^We downgraded the certainty of evidence by two levels for serious imprecision; the CI included both substantial harm and small/no benefit. In addition, the number of events was small (*n* = 58 events)

^o^We downgraded the certainty of evidence by one level for serious imprecision; the CI included both substantial harm and trivial benefit

^p^We downgraded the certainty of evidence by one level for serious indirectness, the ROSE Trial which contributed to 55% of the weight in the analysis for this subgroup, included patients with ARDS and P/F > 120 not 100

^q^We downgraded the certainty of evidence by one level for serious imprecision; the CI included both substantial benefit and small harm

^r^We downgraded the certainty of evidence by one level for serious inconsistency; although the *I*^2^=0% the Forest plot showed that the results of the ROSE Trial are inconsistent with the results of other trials

^s^We downgraded the certainty of evidence by one level for serious indirectness, the ROSE Trial which contributed to 81% of the weight in the analysis for this subgroup, included patients with ARDS and P/F < 120 not only < 100

^t^We downgraded the certainty of evidence by two levels for very serious inconsistency; the *I*^2^ = 65%

^u^We downgraded the certainty of evidence by one level for serious imprecision; the number of events was small and the confidence interval although did not include 1, it included substantial variation in benefit

^v^Although *I*^2^ = 34%, we did not downgrade for inconsistency

^w^We downgraded the certainty in the evidence by two levels for very serious imprecision; the CI included extreme benefit and harm

^x^We downgraded the certainty of evidence by one level for serious indirectness, the intervention, and control in the ROSE Trial differed from other trials (early NMBA and targeting light sedation)

^y^We downgraded the certainty of the evidence by one level for serious imprecision; the CI included both benefit and harm

^z^We downgraded the certainty in the evidence by one level for serious imprecision; the CI included both trivial and moderate benefit

We performed four pre-planned subgroup analyses to investigate the source of heterogeneity for the primary outcomes (ESM). The subgroup analysis by depth of sedation in the control arm indicated the presence of subgroup difference (*P*-interaction = 0.01, *I*^*2*^ = 84%), showing that the effect estimate for hospital mortality was larger in the subgroup that used deep sedation in the control arm (RR 0.71; 95% CI 0.57, 0.89; *P =* 0.003), compared to light sedation (RR 0.99; 95% CI 0.86, 1.15; *P* = 0.93) (Fig. 2). Due to this subgroup difference indicating a different intervention being studied (paralysis and deep sedation versus light sedation), we performed a post hoc sensitivity analysis without the ROSE trial. The results of other subgroup analyses can be found in the (ESM). We were not able to conduct subgroup analyses by proning intervention, PEEP level, or cause of ARDS because of lack of subgroup data, hopefully these important subgroup analyses can be assessed in a future individual patients’ data meta-analysis.

In the post hoc analysis excluding the ROSE trial, 6 trials (592 patients) reported on mortality. The use of NMBA infusion was associated with lower 28-day mortality (RR 0.65; 95% CI 0.51–0.84; *I*^*2*^ = 0%; low certainty; *P <* 0.001) and hospital/90-day mortality (RR 0.71; 95% CI 0.57–0.89; *I*^*2*^ = 0%; low certainty; *P =* 0.003). In addition, NMBA infusion reduced the risk of barotrauma (RR 0.43; 95% CI 0.20–0.90, *I*^*2*^ = 0%; low certainty; *P =* 0.02) and increased VFD at 28 days (MD 1.91; 95% CI 0.28, 3.55, *I*^*2*^ = 0%; low certainty; *P =* 0.02).

Table 2 summarizes the certainty of evidence for each outcome. Overall, we judged the certainty of evidence as moderate or low for 90-day mortality. The certainty of evidence was moderate for barotrauma, and for ICU-acquired weakness, but low for other outcomes.

## Discussion

This updated systematic review and meta-analysis included 7 trials (*n* = 1598). For mortality, we found significant clinical and statistical heterogeneity that precluded meta-analysis of all trials. It appears that a 48-h infusion of NMBA in moderate-to-severe ARDS probably improves 90-day mortality when compared to using a deep sedation strategy, but has no important effect on 90-day mortality when compared to a lighter sedation strategy. A 48-h infusion of NMBA reduces the risk of barotrauma compared to no infusion; however, it did not affect the duration of mechanical ventilation, VFD, ICU length of stay, ICU-acquired weakness, adverse events, and long-term outcomes.

The included studies need to be interpreted in the appropriate methodological context as sedation type and depth, PEEP strategy, and time to enrolment differed. First, all studies, with the exception of the ROSE trial, used deep sedation in both arms which was frequently used in that era; the control arm in the ROSE trial received lighter sedation. This raises the question of unequal comparators across the included studies as evidence suggests that deep sedation may be associated with increased mortality [24]. Second, all the included studies used a low PEEP strategy except for the ROSE trial, which used a higher PEEP strategy (12.6 cm H_2_O in ROSE vs 10.2 cm H_2_O on average in all other included studies). Third, patients were enrolled very early in the ROSE trial with a median time to enrolment of 7.6 h (3.7–15.6 h) versus 16 h (6–29 h) in ACURASYS [9, 12].

Given these differences, the ROSE trial may be too dissimilar to pool with the other studies. In the subgroup analysis by depth of sedation, the effect estimate for hospital mortality was larger in the subgroup that used deep sedation in the control arm (RR 0.72; 95% CI 0.58, 0.91), compared to light sedation (i.e., only the ROSE trial). NMBA infusion with deep sedation may be superior to deep sedation without paralysis, but not better than light sedation alone. The results of the ROSE trial diverged from the results of published studies. One possible explanation is the difference in control arms (light sedation vs deep sedation) or the timing of use of NMBA (early vs late). Other factors may have led to inconsistency in the results and future individual patient data meta-analysis could help shed some light on these issues. Lastly, it is possible that the benefit observed in older trials with NMBA arm may not be due to the use NMBA, but instead a detrimental effect of deep sedation in the control arm.

A recent meta-analysis on this topic drew a different conclusion [25]. Hua et al. reported that an NMBA infusion in ARDS reduces 21- and 28-day mortality, barotrauma, and improves oxygenation at 48 h. However, authors only included 6 of the 7 trials, thus less comprehensive. Furthermore, authors did not address the statistical heterogeneity of their results; therefore, relaying a message that NMBA infusion is beneficial in any context, which we consider an inappropriate interpretation of the literature.

Similar to the findings of our previously published review [8], we found a significant, and more precise, reduction in barotrauma with the use of NMBA infusion. There are several physiologic hypotheses for this finding. One possible mechanism is by minimizing swings in transpulmonary pressures with spontaneous breathing, known as the Pendelluft phenomenon [26, 27]. An additional mechanism is by improving patient-ventilator synchrony. Studies that assessed ventilator dyssynchrony using an esophageal balloon showed that the use of NMBA improves patient-ventilator interaction [22, 28].

In this meta-analysis, the pooled data demonstrate improvement in oxygenation as shown by the increasing PaO_2_/FiO_2_ ratio at 72 h post-randomization. The change is modest and may not be clinically important. However, a careful review of previous studies suggests that the maximum increase in PaO_2_/FiO_2_ happens in day 5–7 which might explain the incremental increase in PaO_2_/FiO_2_ at 72 h [29, 30]. The notion that NMBAs improve hypoxemia among those with moderate to severe ARDS has been supported by clinical studies but the mechanism remains unclear [19, 29]. Some of the proposed mechanisms include improved ventilator synchrony, decreased work of breathing, facilitation of lung protective strategy, better lung recruitment, and improved lung compliance.

With regards to the safety of NMBA infusion use, this review did not detect an increase in the rate of ICU-acquired weakness with NMBA infusion (RR 1.16; 95% CI 0.98, 1.37). The incidence of ICU-acquired weakness in ARDS patients, in general, approximates 36% [31]. Previous studies failed to show a clear association between NMBA use and the development of ICU-acquired weakness [32]. Moreover, the use of corticosteroids, a clear confounder for ICU-acquired weakness, was similar in both groups among the included studies. This re-assuring result could also be explained by the fact that NMBAs were used for a short duration in all of these studies.

Recently, several reviews were published on this topic [25, 33, 34]. However, all have ignored the statistical and clinical heterogeneity between trials and performed meta-analysis of all trials combined. Therefore, yielding potentially misleading conclusions. In addition, most published reviews missed relevant trials.

This review has noteworthy strengths including adherence to a review protocol, a comprehensive literature search, duplicate independent judgements about study eligibility and risk of bias, inclusion of non-English published studies, and the use of subgroup analyses to test the robustness of the data.

There are several important limitations of the results. First, we were unable to assess for publication bias because of the limited number of included studies. Second, the open-label design in three studies may have also influenced the measurement of secondary outcomes. Third, two of the included studies, contributing 12.8% of the weight in this meta-analysis, used vecuronium instead of cisatracurium; an observational study found cisatracurium to be associated with better outcomes [35]. Lastly, as with any subgroup analysis, the results should interpreted with great caution, we can only speculate about why the results of the ROSE trial differed from prior trials, as many factors, measured or unmeasured, may have influenced the results.

## Conclusion

In summary, this review suggests that the impact of NMBA infusion on mortality depends on the strategy used in the control arm, showing reduced mortality when compared to deep sedation, but no effect on mortality when compared to lighter sedation. Although, NMBAs reduce barotrauma, their effect on other outcomes remains unclear. Future research, including an individual patient data meta-analysis, could help clarify some of the observed findings in this updated systematic review.

## Supplementary information

**Additional file 1:** **Table S1:** Search Strategy. **Table S2:** Risk of Bias Assessment. **Table S3:** Subgroup Analyses for Hospital Mortality outcome. **Figure S1:** Pooled mortality outcome. **Figure S2:** Duration of Mechanical Ventilation. **Figure S3:** Oxygenation at 24, 48, and 72 hours. **Figure S4:** Hospital Mortality subgroup analysis by severity of ARDS. **Figure S5:** Hospital Mortality subgroup analysis by risk of bias.

**Additional file 2.** PRISMA 2009 checklist.

## Data Availability

Not applicable.
