# Supplementary material for: Neuromuscular blocking agents in acute respiratory distress syndrome: updated systematic review and meta-analysis of randomized trials
Source: Intensive Care Med Exp. 2020 Oct 23;8:61. doi: 10.1186/s40635-020-00348-6 (PMC7582438; doi:10.1186/s40635-020-00348-6)
Supplement: Supplementary file 1 — Additional file 1: Table S1: Search Strategy. Table S2: Risk of Bias Assessment. Table S3: Subgroup Analyses for Hospital Mortality outcome. Figure S1: Pooled mortality outcome. Figure S2: Duration of Mechanical Ventilation. Figure S3: Oxygenation at 24, 48, and 72 hours. Figure S4: Hospital Mortality subgroup analysis by severity of ARDS. Figure S5: Hospital Mortality subgroup analysis by risk of bias. [file 40635_2020_348_MOESM1_ESM.docx]

**Neuromuscular blocking agents in acute respiratory distress syndrome: updated systematic review and meta-analysis**

**Authors**

Nehal Tarazan, Moayad Alshehri, Sameer Sharif, Zainab Al Duhailib, Morten Hylander Moller, Emilie Belley-Cote, Muhammed Alshahrani, Bandar Baw, Maureen Meade, Waleed Alhazzani, for the GUIDE Group

**Table of Contents:**

**Table S1:** Search Strategy

**Table S2:** Risk of Bias Assessment

**Table S3:** Subgroup Analyses for Hospital Mortality outcome

**Figure S1:** Pooled mortality outcome

**Figure S2:** Duration of Mechanical Ventilation

**Figure S3:** Oxygenation at 24, 48, and 72 hours

**Figure S4:** Hospital Mortality subgroup analysis by severity of ARDS

**Figure S5:** Hospital Mortality subgroup analysis by risk of bias

**Table S1. Search Strategy**

1     exp lung diseases/

2     Respiratory Distress*.mp.

3     adult Respiratory Distress Syndrome.mp.

4     ARDS.mp.

5     idiopathic respiratory distress [syndrome.mp](http://syndrome.mp/).

6     transfusion related acute lung [injury.mp](http://injury.mp/).

7     shock [lung.mp](http://lung.mp/).

8     human [ards.mp](http://ards.mp/).

9     noncardiogenic pulmonary [edema.mp](http://edema.mp/).

10     increased-permeability pulmonary [edema.mp](http://edema.mp/).

11     stiff [lung.mp](http://lung.mp/).

12     acute respiratory [distress.mp](http://distress.mp/).

13     (acute lung injury or ALI).mp.

14     [pneumonia.mp](http://pneumonia.mp/).

15     hyaline membrane [disease.mp](http://disease.mp/).

16     exp respiratory insufficiency/

17     (respiratory adj1 (failure or insufficiency)).mp.

18     or/1-17

19     exp Neuromuscular Blocking Agents/

20     Neuromuscular Blocking Agent*.mp.

21     Neuromuscular Blocker*.mp.

22     curare like [activity.mp](http://activity.mp/).

23     ((curariform or curarizing) adj1 agent*).mp. [mp=protocol supplementary concept, rare disease supplementary concept, title, original title, abstract, name of substance word, subject heading word, unique identifier]

24     Neuromuscular Blocking drug*.mp.

25     (Rapacuronium or Raplon or Mivacurium or Mivacron or Atracurium or Tracrium or Doxacurium or Nuromax or Cisatracurium or Nimbex or Vecuronium or Norcuron or Rocuronium or Zemuron or Pancuronium or Pavulon or Tubocurarine or Jexin or gallamine or Flaxedil or Pipecuronium or alcuronium orcurare or toxiferine).mp.

26     or/19-25 (

27     randomized controlled [trial.pt](http://trial.pt/).

28     Randomi?ed Controlled trial*.mp.

29     Randomi?ed clinical trial*.mp.

30     Random Allocation/

31     Random [allocation.mp](http://allocation.mp/).

32     random*.tw.

33     clinical trial/

34     controlled clinical trial/

35     single-blind method/

36     double-blind method/

37     ((singl* or doubl* or trip* or trebl*) adj25 (blind* or mask*)).mp.

38     Placebos/

39     placebo*.tw.

40     drug therapy.fs.

41     trial.ab.

42     groups.ab.

43     or/27-42

44     humans/

45     animals/

46     45 not (44 and 45)

47     43 not 46

48     18 and 26 and 47

**Reference list of excluded articles**

Observational studies (6)

1. Cawley, M., Czosnowski, Q., & Palkovic, L. (2010). Comparison of sedatives, analgesics and neuromuscular blocker requirements during pressure control and airway pressure release ventilation. *Critical Care Medicine.Conference: 40th Critical Care Congress of the Society of Critical Care Medicine San Diego, CA United States.Conference Start: 20110115 Conference End: 20110119.Conference Publication: (Var.Pagings), 38*, A251.
2. Conti, G., Vilardi, V., Rocco, M., DeBlasi, R. A., Lappa, A., Bufi, M., et al. (1995). Paralysis has no effect on chest wall and respiratory system mechanics of mechanically ventilated, sedated patients. *Intensive Care Medicine, 21*(10), 808-812.
3. Kallet, R. H., Eisner, M., & Luce, J. M. (2001). Sedation and neuromuscular blocking agent (NMBA) requirements during initiation of low vo ventilation in patients with acute lung injury (ALI). *Respiratory Care, 46*(10), 1122.
4. Lee Hough, C. (2006). Neuromuscular sequelae in survivors of acute lung injury. *Clinics in Chest Medicine, 27*(Acute Respiratory Distress Syndrome.), 691-703.
5. Lefrant, J.-Y. ; Cuvillon, P. ; Pandolfi, J.-L. Continuous infusion of atracurium in critically ill patients with acute respiratory distress syndrome (ARDS). European Society of Anaesthesiologists 1997; british journal of anaesthesia 1997, 78 (SUPP 1): A.363.
6. Calon, B. ; Launoy, A. ; Pottecher, T. Long-term use of rocuronium in ICU patients with or without multi organ failure. European Society of Anaesthesiologists 1999; british journal of anaesthesia 1999, 82 (SUPP 1): A.547.

Review Articles (8)

1. Behbehani, N. A., Al-Mane, F., D'yachkova, Y., Pare, P., & FitzGerald, J. M. (1999). Myopathy following mechanical ventilation for acute severe asthma: The role of muscle relaxants and corticosteroids. *Chest, 115*(6), 1627-1631.
2. De Jonghe, B., Lacherade, J. -., Durand, M. -., & Sharshar, T. (2007). Critical illness neuromuscular syndromes. *Critical Care Clinics, 23*(Early Mobility of the ICU Patient.), 55-69.
3. Elsasser, S., Schachinger, H., & Strobel, W. (1999). Adjunctive drug treatment in severe hypoxic respiratory failure. *Drugs, 58*(3), 429-446. Emery, E. R. (1971). [Use of pancuroniumbromide in the intensive care unit]. [Der Gebrauch von Pancuroniumbromid in der Intensivstation.] *Anaesthesist, 20*(6), 237-238.
4. Forel, J. M., Roch, A., & Papazian, L. (2009). Paralytics in critical care: Not always the bad guy. *Current Opinion in Critical Care, 15*(1), 59-66.
5. Frank, A. J., & Thompson, B. T. (2010). Pharmacological treatments for acute respiratory distress syndrome. *Current Opinion in Critical Care, 16*(1), 62-68.
6. Freebairn, R., & McHugh, G. (2010). Neuromuscular blockade in the optimal management of mechanical ventilation of patients with respiratory distress. *Current Respiratory Medicine Reviews, 6*(4), 223-228.
7. Horner, D., & Cairns, C. (2011). Early neuromuscular blockade in severe ARDS. *Journal of the Intensive Care Society, 12*(2), 153-154.
8. Lagneau, F. (2008). Indications and uses of neuromuscular blocking agents in the ICU. [Indications et utilisation des curares en reanimation.] *Annales Francaises d'Anesthesie Et De Reanimation, 27*(7-8) (pp 567-573), ate of Pubaton: Juy 2008/August 2008.

Systematic review or meta-analysis (1)

1. Cools, F., & Offringa, M. (2009). Neuromuscular paralysis for newborn infants receiving mechanical ventilation. *Cochrane Database of Systematic Reviews, 4) , 2009*, ate of Pubaton: 2009.

Duplicates (1)

1. Papazian, L., Forel, J. -., Gacouin, A., Perrin, G., Jaber, S., Arnal, J. -., et al. (2009). Systematic two-day muscle relaxants course in the early phase of severe acute respiratory distress syndrome. A multicenter randomized controlled trial. *Intensive Care Medicine.Conference: 22nd Annual Congress of the European Society of Intensive Care Medicine, ESICM Vienna Austria.Conference Start: 20091011 Conference End: 20091014.Conference Publication: (Var.Pagings), 35*, S6.

Editorial or letter (6)

1. Devlin, J. W., Garpestad, E., & Hill, N. S. (2010). Neuromuscular blockers and ARDS. *New England Journal of Medicine, 363*(26), 2562-4.
2. Gusmao, D. (2010). Neuromuscular blockers and ARDS. *New England Journal of Medicine, 363*(26), 2562-2563.
3. Sevransky J. ACP Journal Club. 48 hours of cisatracurium reduced 90-day mortality in patients with early, severe ARDS. *Ann Intern Med. 2011 Jan 18;154(2):JC1-3.*
4. Pathak, V., & Islam, T. (2011). Neuromuscular blockers improves outcome in severe and early adult respiratory distress syndrome. *Clinical Pulmonary Medicine, 18*(2), 95.
5. Puthucheary, Z., Hart, N., & Montgomery, H. (2010). Neuromuscular blockers and ARDS. *New England Journal of Medicine, 363*(26), 2563.
6. Slutsky, A. S. (2010). Neuromuscular blocking agents in ARDS. *New England Journal of Medicine, 363*(12), 1176-1180.

Different intervention or different population (5)

1. Caldwell, J. E., Lau, M., & Fisher, D. M. (1995). Atracurium versus vecuronium in asthmatic patients: A blinded, randomized comparison of adverse events. *Anesthesiology, 83*(5), 986-991.
2. de Lemos, J. M., Carr, R. R., Shalansky, K. F., Bevan, D. R., & Ronco, J. J. (1999). Paralysis in the critically ill: Intermittent bolus pancuronium compared with continuous infusion. *Critical Care Medicine, 27*(12), 2648-2655.
3. Farenc, C., Lefrant, J. Y., Audran, M., & Bressolle, F. (2001). Pharmacokinetic-pharmacodynamic modeling of atracurium in intensive care patients. *Journal of Clinical Pharmacology, 41*(1), 44-50.
4. Farenc, C., Lefrant, J. -., Audran, M., Saissi, G., De La Coussaye, J. -., & Bressolle, F. (2000). Pharmacokinetics of atracurium and laudanosine in intensive care patients with acute respiratory distress syndrome undergoing mechanical ventilation. *Clinical Drug Investigation, 19*(2), 143-150. Forel, J. -., Roch, A., Marin, V., Michelet, P., Demory, D., Blache, J. -., et al. (2006). Neuromuscular blocking agents decrease inflammatory response in patients presenting with acute respiratory distress syndrome. *Critical Care Medicine, 34*(11), 2749-2757.
5. Hadbavny, A.; Rafkin, H.; Hoyt, J. Rocuronium infusions in the intensive care unit. European congress; 9th (1996; Sep : Glasgow). Intensive care medicine. 1996, 22 (3): 835-838

**Table S2** **Risk of Bias Assessment**

| **Study** | **Sequence generation** | **Allocation concealment** | **Blinding** | **Withdrawal; loss to follow-up** | **Selective outcome reporting** | **Free of other bias** | **Overall Risk of Bias** |
| --- | --- | --- | --- | --- | --- | --- | --- |
| Gainnier  2004 | Low risk  Computer-generated random number sequences | Low risk  Centralized | Low risk  Nurses aware of assignment; infusion covered by sheet. | Low risk  None | Low risk  None | Low risk  None | Low |
| Forel  2006 | Low risk  Computer-generated random number sequences | Low risk  Centralized | Low risk  Nurses aware of assignment; infusion covered by sheet. | Low risk  None | Low risk  None | Low risk  None | Low |
| Papazian  2010 | Low risk  Computer-generated random number sequences. | Low risk  Centralized, using undisclosed block sizes. | Low risk  Blinding of patients, clinicians evaluators, investigators, analysts. | Low risk  None | Low risk  None | Low risk  None | Low |
| Lyu  2014 | Low risk | Unclear risk  Not reported | High risk  Probably only patients blinded. | Unclear risk  Not reported unlikely blinded, no such attempt. | High risk  Reported those mentioned in methods, others they did not report, maybe they did not look for. | High risk  Unclear likely data were switched can lead to analysis errors. | High |
| Rao  2016 | Low risk | Low risk | Unclear risk  Mentioned single blind, likely only patients blinded. | High risk  High risk, 19% vs 17% randomized patients were excluded, then 7 were crossed over, no data reported for the 7 patients who crossed over | Low risk | Unclear risk  6 crossover patients in the abstracts but 7 in figure 1 and main results | High |
| Guervilly  2016 | Low risk | Low risk | Low risk | Low risk | Low risk | Low risk | Low |
| ROSE  2019 | Low risk | Low risk  Process Not specifically explained | Low risk  -Primary end point unblinded. -uncertainty about In-hospital assessors of end points being unaware of treatment group, but all post-discharge end points were assessed by trial personnel who were unaware of the group assignment. | Low risk | Low risk | Low risk  Although trial was topped early for futility it included a very large number of events | Low |

**Table S3.** **Subgroup Analyses for Hospital Mortality outcome**

| **Subgroup** | **Number of patients (n)** | **Relative risk (95% CI)** | **P- interaction** | **I^2^** |
| --- | --- | --- | --- | --- |
| Depth of sedation in control arm  Deep sedation  Light sedation | 472  1006 | 0.71 (0.57, 0.89)  0.99 (0.86, 1.15) | 0.01 | 84% |
| Methodologic quality of trials  Low risk of bias  High/Unclear risk of bias | 1345  133 | 0.91 (0.80, 1.02)  0.28 (0.06, 1.29) | 0.13 | 55.5% |
| Cause of ARDS | Unable to do it due to lack of data in the recent trials | | | |
| PaO_2_/FiO_2_  > 100  ≤ 100 | 542  495 | 0.87 (0.71-1.06)  0.95 (0.82-1.11) | 0.47 |  |
| Duration of infusion | All trials used 48 hours infusion | | | |
| PEEP Strategy | Not feasible, requires individual patient data | | | |
| Timing of initiation of NMBA  Early  Late | 472  1006 | 0.72 (0.58, 0.91)  0.99 (0.86, 1.15) | 0.02 | 81.6% |
| Prone positioning | Not feasible, requires individual patient data | | | |

ARDS: acute respiratory distress syndrome; PaO_2_/FiO_2_: ratio of partial arterial pressure of oxygen to fraction of inspired oxygen; PEEP: Peak end expiratory pressure


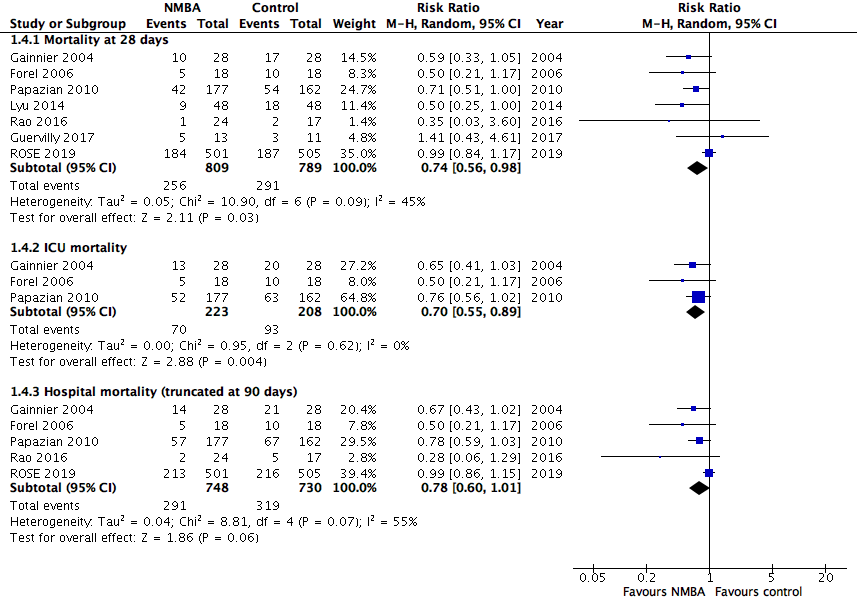


**Fig. S1** Forest plot comparing neuromuscular blockers and placebo or usual care for the following outcomes: 28 days, ICU, and hospital (truncated at 90 days); results are shown by using random-effects model with relative risk and 95% confidence interval.


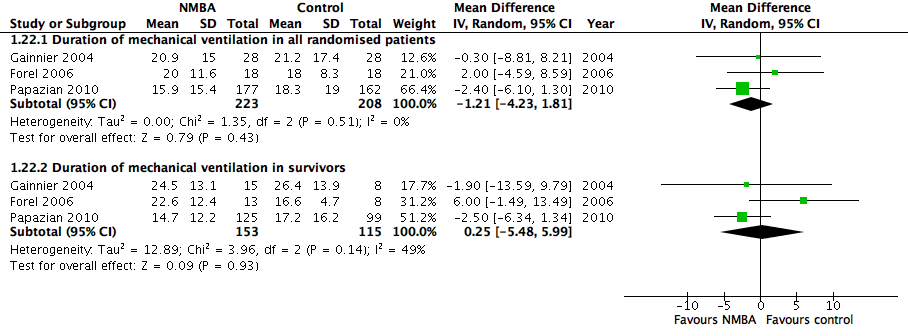


NMBA: Neuromuscular blocking agents; CI: confidence interval

**Fig. S2** Forest plot comparing neuromuscular blockers and placebo for the duration of mechanical ventilation in all patients and in survivors; results are shown by using random-effects model with relative risk and 95% confidence interval


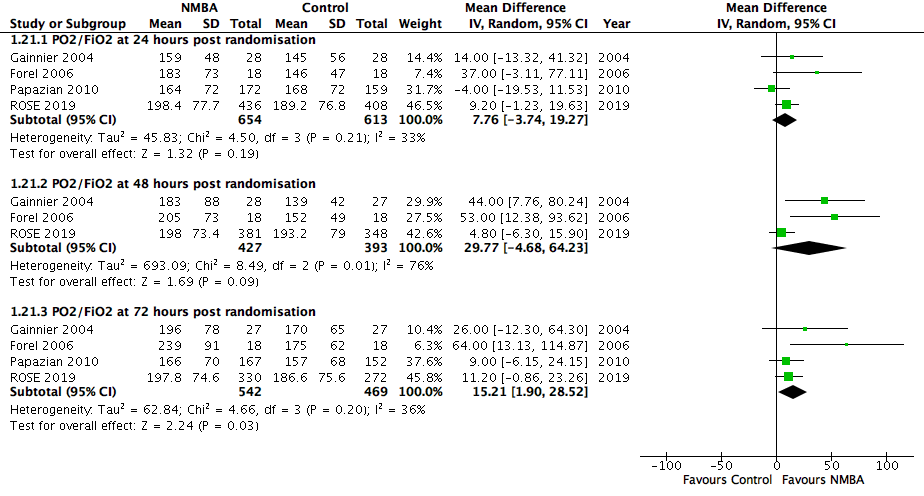


NMBA: Neuromuscular blocking agents; CI: confidence interval

**Fig. S3** Forest plot comparing neuromuscular blockers and placebo for the oxygenation outcome (measured by using PaO_2_/FiO_2_ at 24 to 72 hours after randomization); results are shown by using random-effects model with mean difference and 95% confidence interval


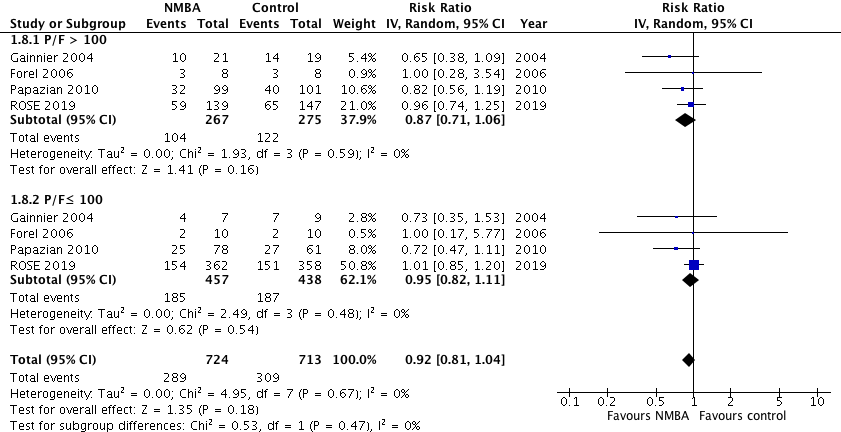


NMBA: Neuromuscular blocking agents; CI: confidence interval; PaO_2_/FiO_2_: ratio of partial arterial pressure of oxygen to fraction of inspired oxygen

**Fig. S4** Forest plot showing subgroup analysis for hospital mortality outcome by severity of hypoxemia at baseline (according to PaO_2_/FiO_2_ ratio); results are shown by using fixed-effect model with risk ratio and 95% confidence interval, P-interaction=0.47, indicating no subgroup effect.


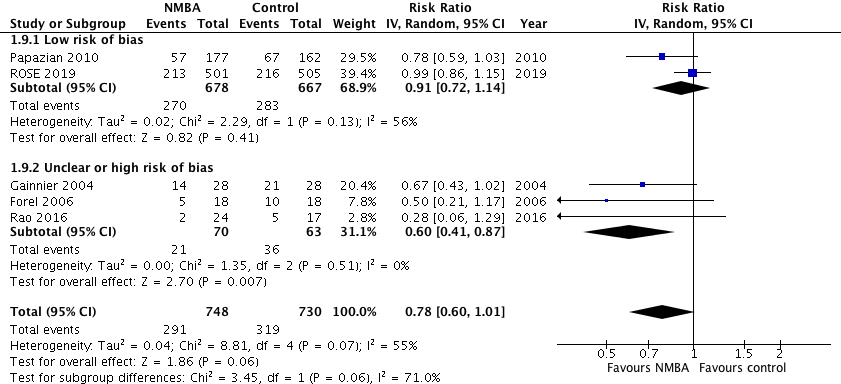


NMBA: Neuromuscular blocking agents; CI: confidence interval

**Fig. S5** Forest plot showing subgroup analysis for hospital mortality outcome by risk of bias (low risk of bias vs other); results are shown by using random-effects model with risk ratio and 95%
